# Preparation and Characterization of Celecoxib Dispersions in Soluplus^®^: Comparison of Spray Drying and Conventional Methods

**Published:** 2015

**Authors:** Alireza Homayouni, Fatemeh Sadeghi, Ali Nokhodchi, Jaleh Varshosaz, Hadi Afrasiabi Garekani

**Affiliations:** a *Department of Pharmaceutics, School of Pharmacy, Mashhad University of Medical Sciences, Mashhad, Iran. *; b *Targeted Drug Delivery Research Center, School of Pharmacy, Mashhad University of Medical Sciences, Mashhad, Iran. *; c *Chemistry and Drug Delivery Group, Medway School of Pharmacy, University of Kent, ME4 4TB, Kent, United Kingdom. *; d *Drug Applied Research Center and Faculty of Pharmacy, Tabriz University of Medical Sciences, Tabriz, Iran. *; e *Department of Pharmaceutics, Faculty of Pharmacy and Novel Drug Delivery System Research Center, Isfahan University of Medical Sciences, Isfahan, Iran. *; f *Pharmaceutical Research Center, School of Pharmacy, Mashhad University of Medical Sciences, Mashhad, Iran.*

**Keywords:** Celecoxib, Solid dispersion, Soluplus^®^, Dissolution rate, Solid states

## Abstract

The present study deals with characterization of dispersions of a poorly water-soluble drug, celecoxib (CLX) in polyvinyl caprolactame–polyvinyl acetate–polyethylene glycol graft copolymer (Soluplus^®^ (SOL)) prepared by different techniques.

Dispersions of CLX in SOL at different ratios (2:1, 1:1, 1:2, 1:4 and 1:6) were prepared by spray drying, conventional solvent evaporation and melting methods. The solid states of samples were characterized using particle size measurements, optical and scanning electron microscopy, XRPD, DSC and FT-IR. The Gordon-Taylor equation was used to predict the T_g_ of samples and the possibility of interaction between CLX and SOL. The solubility and dissolution rate of all samples were determined. Stability of samples was studied at ambient conditions for a period of 12 months.

DSC and XRPD analyses confirmed amorphous state of drug in samples. Surprisingly dispersions of CLX:SOL with the ratio of 2:1 and 1:1 showed slower dissolution rate than CLX while other samples showed higher dissolution rate. At 1:2 ratio the spray dried samples exhibited higher dissolution rate than corresponding samples prepared by other methods. However at higher SOL content (1:4 and 1:6), samples prepared by different methods showed similar dissolution profiles. The stability studies showed that there were no remarkable changes in the dissolution profiles and solid state of the drug after 12 months storage at ambient conditions.

It was concluded that SOL was a proper carrier to enhance the dissolution rate of CLX. At high SOL ratios the method of preparation of dispersed samples had no effect on dissolution rate, whilst at low SOL content spray drying was more efficient method.

## Introduction

The oral route of drug administration is the most common and preferred method of delivery due to convenience and ease of ingestion. The poor solubility and slow dissolution rate of an active pharmaceutical ingredient are main drawbacks for the pharmaceutical industry to develop a suitable dosage form. At present approximately 40% of new chemical entities identified by pharmaceutical companies are poorly water-soluble (1) and their bioavailability and the extent of absorption are limited by poor solubility in water (2). Various techniques such as solid dispersions (3-6), preparation of liquisolid (7, 8), making salts (9) have been used to overcome these limitations. The dispersion of one or more active ingredients at solid state in an inert crystalline or amorphous carrier (generally water soluble) such as PEG, PVP or sugars, prepared by the melting (fusion), solvent or melting-solvent method (3, 10) is one of the most promising strategies to improve the oral bioavailability of poorly water soluble drugs such as itraconazol, indomethacin, *etc* (4, 5). In these systems formation of amorphous state, reduction in drug particle size and improvement in drug wettability are contributing in enhancement in bioavailability of drug has been widely used.

Despite the long history of using these dispersions in pharmaceutical products, the multiplicity of polymeric carriers that have been used is still limited. Polyvinyl caprolactam–polyvinyl acetate–polyethylene glycol graft copolymer (Soluplus^®^), a new polymer with amphiphilic properties is the novel excipient which is used as a carrier matrix and solubilizer. 

This polymer is playing the role of both carrier and active solubilizer (through micelle formation in water) and dispersion of drug in this carrier could be considered as the fourth generation of solid dispersions (5). Because of low T_g_ value (approximately 70 °C), SOL could be used in both solvent evaporation and melting method (11) for production of dispersions. Improvement in dissolution rate of several active pharmaceutical ingredients (APIs) in aqueous media was investigated by Soluplus^®^ using extrusion techniques (12-14). *In-vivo* studies reported by Linn *et al*. have shown an enhancement in bioavailability of danazol, fenofibrate and itraconazol (BCS class II drugs) (13). Similar results have been reported by nanosized bicalutamide with Soluplus^®^ (15). 

CLX, is a nonsteroidal anti-inflammatory drug (NSAID) that is used for the treatment of osteoarthritis, rheumatoid arthritis, and management of pain. CLX has better efficacy compared to other NSAIDs (*e.g*. naproxen and diclofenac) in these pathophysiological states (16). 

The low aqueous solubility of CLX leads to incomplete oral bioavailability (17). According to the biopharmaceutical classification system (BCS), CLX can be categorized as class II drugs (*i.e*., poor water-solubility and high GI permeability) (18). It has been reported that the bioavailability of CLX is only 30% when given in capsule dosage form in dogs and the amount of drug absorption is limited by dissolution rate (19, 20). Thus it is important to explore a promising technique to enhance the solubility and dissolution rate of this drug without jeopardizing its chemical and solid state stabilities.

Several formulation approaches have been attempted to improve the dissolution behavior of CLX. Examples include formulation of solid dispersions (21-23), complexation with β-cyclodextrins (24), manipulation of the solid state of the drug (25), microemulsions (26, 27), and nanonization with surfactants (17, 19).

Dispersions of CLX using different carriers have been attempted in many studies, however in these studies no attention has been paid on the effect of preparation method of the dispersion systems on dissolution rate of drug. The present work has been performed aiming to study dispersions of (CLX) in new grafted copolymer, Soluplus, prepared by different techniques such as spray drying (SD), conventional solvent evaporation and melting methods. Moreover there is no study in the literature related to the use of SOL as a suitable carrier for preparation of CLX solid dispersions. 

For comparison purposes physical mixtures (PM) of drug-carrier were also prepared. Dispersions were characterized using the DSC, XRPD, FT-IR, saturation solubility and *in-vitro* dissolution tests.

## Experimental

*Materials*

Celecoxib was purchased from Arastoo chemical company (Iran), Soluplus was donated from BASF (Germany) and sodium dodecyl sulfate was from Merck (Germany). All other solvents and chemicals were of analytical grade used as obtained.

*Preparation of dispersions of CLX in SOL*

Dispersions of CLX (CLX) in Soluplus (SOL) were prepared using three different approaches at weight ratios of 2:1, 1:1, 1:2, 1:4 and 1:6 (CLX:SOL) as follows.

*Spray drying method*

In this method, dispersions were prepared by spray drying of methanolic solutions of CLX and SOL in specified mass ratios with an overall concentration of 5% (w/v) in a Büchi Mini Spray Dryer B290, Inert Loop B-295 (Büchi Labortechnik AG, Flawil, Switzerland). The spray drying was performed with the following conditions: inlet temperature 80 °C, outlet temperature 50 °C, solution flow rate 5 mL/min and N_2_ flow rate 35 m^3^/h. The spray dried samples were stored in a desiccator until used for further studies.

*Solvent evaporation method*

Methanol was used as a solvent because of high solubility of CLX in methanol (114 mg/mL). The required amounts of CLX and carrier were dissolved in minimum amount of methanol to obtain clear viscous solution. The viscous solution obtained from drug/carrier mixture could prevent mobilization, separation and finally crystallization of saturated drug while these events may occur when the solution has low viscosity. The solvent was removed at 40 °C in an oven until complete drying of samples. The dispersions were then pulverized using a mortar and pestle, passed through 60-mesh sieve (250 µm) and stored in a desiccator until use for further studies.

*Melting method*

This type of dispersion was prepared by heating accurately weighed amounts of SOL and CLX in a beaker in a liquid paraffin bath at 130 5 °C. The mixtures were stirred using glassy spatula continuously and after 10 min the pasty mixture was cooled to reach room temperature. The dispersions were then pulverized using a mortar and pestle, passed through 60-mesh sieve (250 µm) and stored in a desiccator until use for further studies.

*Preparation of physical mixtures*

The physical mixtures of CLX and SOL were prepared by mixing (sieved fractions) the both components using mortar and pestle. 

*Preparation of amorphous CLX*

Amorphous CLX was prepared by melting the pure CLX at 170 C under nitrogen atmosphere and then rapidly quench cooling the molten sample with an ice bath as described by Andrews *et al*. (22). The prepared amorphous CLX was used for DSC analysis. 

*Characterization of samples*

*Determination of spray drying yield*

The yields of spray dried samples were calculated by determining the weight of recovered particles divided by the total original weight of CLX and SOL.

*Particle size analysis*

Particle size measurement of sieved fractions of samples prepared by conventional solvent evaporation and melting method was performed by optical microscopy (Olympus model BX60, Japan). To do this, small amounts of each sample was spread on glass slide and Martins diameter of minimum 100 particles was measured by Dinolite digital microscope software Ver. 3.3.016 and mean particle size of each sample was determined. Dynamic laser light scattering technique (Nano-Zetasizer, Malvern, UK) was used for particle size determination of pure CLX and spray dried samples. To estimate mean particle size of pure CLX and spray dried samples approximately 2 mg of each powder sample was dispersed in 2 mL of deionized water (kept 1 min in ultrasound water bath) and then subjected to Zetasizer. The measurements were performed in triplicate to determine the z-average size of the particles.

*Determination of saturation solubility*

The saturation solubility measurements were performed on pure CLX, physical mixtures and dispersed samples. Samples of 5 mg were added to 25 mL double-distilled water which was shaken at 100 rpm in an air bath (25 °C) for 24 h, then the resulting suspensions was filtered through a 0.22 μm filter. Concentration of CLX was determined spectrophotometrically at 253 nm. The solubility of each sample was determined in triplicates and the mean values and standard deviations were reported.

*Morphological analysis*

The morphologies of pure untreated CLX and spray dried samples were examined using a scanning electron microscope (LEO 1450 VP, Germany) at an acceleration voltage of 20 kV. Samples were coated with a thin gold-palladium layer by sputter coater (SC 7620, England). Optical microscopy (Olympus BX-60, Japan) was also used to investigate the morphology of samples prepared by conventional methods. 

*Differential scanning calorimetry (DSC)*

Calorimetric studies were performed using a DSC 822e Mettler-Toledo (Mettler Toledo, Switzerland) equipped with a refrigerated cooling system. Samples of pure CLX (both crystalline and amorphous), dispersed samples and physical mixtures (3–5 mg) were placed in aluminum pans sealed with a lid. The DSC was calibrated using indium standard. The crimped aluminum pans were heated from 20 to 200 °C at a scanning rate of 10 °C/min under nitrogen atmosphere. Onset temperatures and melting points of the samples were automatically calculated using the software provided (STARe Ver. 10.00 Mettler Toledo, Switzerland).

*Gordon-Taylor calculations*

The Gordon-Taylor equation shown below, was used to predict the theoretically T_gmix_ of binary solid dispersion samples. These values were compared to the experimentally determined T_g_. 

$T_{\mathrm{gmix}}=\frac{w_{1}T_{\mathrm{g1}}+{\mathrm{kw}}_{2}T_{\mathrm{g2}}}{w_{1}+{\mathrm{kw}}_{2}}$

$K\approx \frac{T_{\mathrm{g1}}\rho_{1}}{T_{\mathrm{g2}}\rho_{2}}$

T_g1_ and T_g2_ are the glass transition temperatures, and w_1_ and w_2_ are the weight fractions of the two components. K is estimated from the true density (ρ) and T_g_ of pure CLX and SOL. The true densities of SOL and amorphous CLX were determined to be 1.03 and 1.35 g/cm^3^, respectively. 

*X-ray powder diffraction studies (XRPD)*

X-Ray powder diffraction patterns were obtained for selected samples using a Bruker, D8 Advance, Germany diffractometer, with Cu Kα radiation (λ= 1.54Ả). The diffraction pattern was measured with a voltage of 30 kV and a current of 40mA in the range of 5°-40° (2) in a step scan mode.

*Fourier transform infrared spectroscopy (FT-IR)*

The FT-IR spectra of selected samples were obtained using a Perkin Elmer spectrum two FTIR (PerkinElmer, Waltham, MA). Samples were prepared by potassium bromide (KBr) and scanned against a blank KBr disk at wave numbers ranging from 4000 to 450 cm^-1^ with resolution of 1.0 cm^-1^.

*Dissolution studies*

Dissolution studies were carried out in an automated dissolution tester (Pharmatest, Germany) using the USP Apparatus 2 (paddle) method. The paddle speed and bath temperature were set at 50 rpm and 37 °C, respectively. Dissolution medium was 1 liter of distilled water containing 0.25% (w/v) sodium dodecyl sulfate (SLS) (21). An accurate weight of samples equivalent to 40 mg CLX was added directly to the vessels. Samples were taken from the vessels through sintered filter by a peristaltic pump (Alitea, Sweden), and assayed at 253 nm by a multi-cell transport spectrophotometer (Shimadzu, Japan) based on calibration curve obtained for CLX at this wavelength. Each sample was determined in triplicate. 

*Dissolution parameters*

The dissolution efficiency (DE) of a pharmaceutical dosage form is defined as the area under the dissolution curve (y) up to a certain time t, expressed as a percentage of the area of the rectangle described by 100% dissolution in the same time (28).

$\mathrm{DE\%}=\frac{\int_{{}^{\circ}}^{t}\mathrm{y.dt}}{y_{100}\mathrm{.t}}\times 100$

 An alternative parameter that describes the dissolution rate is the mean dissolution time (MDT); MDT was calculated for each formulation using the following equation:

$\mathrm{MDT}=\sum t_{i}.∆M_{i}/\sum ∆M_{i}$

$\bar{t}=(t_{i}+t_{i+1})/2$

$∆M_{i}=(M_{i+1}-M_{i})$

Where t_i_*¯* is the midpoint of the time period during which the fraction *M *of the drug has been released from sample (28).

*Long term stability*

The prepared samples were stored at ambient conditions for a period of 12 months and their stability in solid states and dissolution were investigated by XRPD, DSC and dissolutions studies as described previously. Comparison of dissolution profiles were performed by calculating, the difference factor *(f*_1_*) *and similarity factor *(f*_2_*)* by the following equations:

$f_{1}=\frac{\sum_{t-1}^{n}\left|R_{t}-T_{t}\right|}{\sum R_{t}}\times 100$

$f_{2}=50\log\left\{{\left[1+\frac{1}{n}\sum_{t-1}^{n}{\left(R_{t}-T_{t}\right)}^{2}\right]}^{-0.5}\right\}$

Where *n* is the number of time points, *R*_t_ is the percentage of the reference sample that was dissolved at the time point t, and *T*_t_ is the percentage of the test samples that was dissolved at the time point *t*. If the *f*_1_ value was between 0-15 and the *f*_2_ value was between 50-100, the dissolution profile of two samples was considered equivalent (28).

*Statistical analysis*

Statistical analysis was performed using SPSS software (version 16). The analysis of variance (ANOVA) was used for comparison of the results in different studies. For all of the tests, the differences of P < 0.05 were considered as statistically significant.

## Results and Discussion

CLX was used as a model drug in this study due to low aqueous solubility. Dispersions of CLX in SOL were prepared by different methods in order to improve the dissolution characteristics of CLX. Although many hydrophilic polymers have been used as a carrier in preparation of dispersed systems, but SOL was chosen in this study because of its capability of enhancing both the solubility and absorption of the drugs (13).

Methanol was used as a solvent for preparation of dispersions using solvent evaporation method because of high solubility of CLX in methanol (114 mg/mL) (29). The CLX: SOL combinations at different mass ratios were dissolved in minimum amount of methanol. The volumes of methanol were varied for different formulations. The viscous solution obtained from drug/carrier mixture could prevent mobilization, separation and finally crystallization of saturated drug (30). 

*Particle size measurement*

As it was shown in Table 1 the mean particle size of spray dried samples was smaller and size distribution was narrower than other samples. In spray dried samples the mean particles size generally increased by increasing the SOL ratio. This effect could be due to the increase in viscosity of feeding solution with increase in SOL content. Increasing the viscosity of solution could generate larger droplets and hence larger particles are produced (31). The results showed that there is no significant difference between mean particle size of 2:1, 1:1 and 1:2 SD samples (P> 0.05) whilst 1:4 and 1:6 SD samples exhibited significant difference to other samples (P < 0.05). The mean particle sizes of samples prepared by conventional solvent evaporation and melting method were in the range of 26-46 µm with high standard deviation. There was no significant difference between mean particle size of samples prepared by solvent evaporation and melting methods (P > 0.05). 

**Table 1:** Mean particle size, polydispersity index (PdI), saturation solubility, dissolution efficiency (DE), mean dissolution time (MDT) and difference and similarity factors after 12 month storage (n=3).

**CLX:SOL**	**Mean particle size (µm)**	**PdI**	**Saturation solubility (µg/ml)**	**DE **_90min_ **(%)**	**MDT (min)**	**Difference factor after 12 month (** ***f***_1_ **) **	**Similarity factor after 12 month (** ***f***_2_ **)**	
**Pure CLX**	5.5±0.8	1	3±0.68	42±0.43	18±0.66			
**2:1 PM** 1			3±0.71	31±0.77	28±0.24			
**1:1 PM**			4±0.51	30±0.44	31±0.33			
**1:2 PM**			5±0.37	29±0.36	27±0.41			
**1:4 PM**			6±0.39	32±0.41	27±0.66			
**1:6 PM**			6±0.64	27±0.61	34±0.35			
**2:1 S** 2	26±32		3±0.39	13±0.66	28±0.24			
**1:1 S**	29±37		4±0.57	14±0.99	30±0.41	14		86
**1:2 S**	34±28		4±0.85	67±0.33	17±0.75			
**1:4 S**	37±22		7±0.53	83±0.62	8±0.38	1		86
**1:6 S**	44±20		7±0.77	86±0.73	7±0.62			
**2:1 M** 3	36±41		3±0.24	16±1.05	26±0.32			
**1:1 M**	31±27		4±0.71	18±0.35	25±0.28	7		87
**1:2 M**	41±33		5±0.63	50±0.74	17±0.44			
**1:4 M**	45±22		8±0.43	83±0.82	9±0.62	1		90
**1:6 M**	46±28		7±0.59	85±0.33	7±0.28			
**2:1 SD** 4	3.1±0.3	0.70±0.14	3±0.21	9±0.35	22±0.18			
**1:1 SD**	2.8±0.2	0.43±0.11	5±0.46	23±0.41	21±0.26	9		78
**1:2 SD**	3.1±0.1	0.39±0.08	6±0.35	76±1.82	14±1.22			
**1:4 SD**	6.2±0.4	0.36±0.12	8±0.31	86±0.66	7±0.73	2		84
**1:6 SD**	6.6±0.4	0.35±0.09	8±0.48	87±0.81	5±0.44			

1 Physical mixture,

2 Solvent evaporation method,

3 Melting method, and

4 Spray drying method

*Yield of spray-dried samples*

The yields of spray dried samples were 28±5.4, 40±7.9, 63±8.8, 43±4.4 and 37±3.5% for 2:1, 1:1, 1:2, 1:4 and 1:6 drug: carrier ratio respectively. The yield of the spray drying process increased with increase in SOL concentration in the samples up to 1:2 ratio (P < 0.05). This was probably due to an increase in the viscosity of the solutions and therefore formation of less fine particles during spray drying. In addition, the presence of SOL could induce the adhesion of particles to each other and promote agglomerate formation. These agglomerates have more chances to be trapped in the collector. In solutions containing low amount of SOL, the drug particles could not form aggregates and fine particles might escape from the collector and trap in the waste filter while spray drying. Although the statistical analyses for particle size measurements showed no significant differences amongst 2:1, 1:1 and 1:2 SD samples but according to polydispersity index (PdI) results, by increasing the SOL ratio, PdI become narrower, and the percentage of fine particles decreased. Therefore the yield of SD samples increased by increasing the SOL ratio. In contrary in samples with 1:4 or 1:6 drug: carrier, the yield of spray drying process decreased. This could be due to the sticky behavior of samples with higher amount of SOL. As a result of low T_g_ (approximately 70 °C), SOL becomes soft and sticky at high temperature of spray drier chamber (80 °C) and therefore they had more tendency to attach to the main chamber and cyclone wall as observed visually. This phenomenon might contribute to the reduced yield of samples. 

*Determination of saturation solubility*

Table 1 shows the saturation solubilities of pure CLX, physical mixture and dispersed samples. The results of the solubility studies indicated that pure CLX has very low solubility in water at 25 C (3±0.68 µg/mL). The saturation solubility of CLX: SOL physical mixtures and dispersions were higher than pure CLX. All dispersed samples showed significant differences in their solubilities compared to pure CLX (P < 0.05) with the exception of 2:1 samples. With increasing the concentration of carrier in the physical mixtures and dispersed samples the saturation solubility of CLX was also increased. This could be due to the amphiphilic structure of SOL. The amount of SOL in all samples was higher than critical micelle concentration (CMC) of SOL which has been reported to be 7.6 mg/L (11) hence SOL could form micelles in water and dissolve the CLX molecules. In other words, SOL with carbonyl-amide groups along with the lipophilic chain was able to interact with hydrophobic APIs and finally led to hydration of drug in the aqueous solution. This mechanism has been reported before for description of SOL as a solubilizer (11). The results showed that the method used for preparation of dispersed samples did not have significant effect on solubility of the drug (P> 0.05).This could be due to the high concentration of SOL (higher than CMC) used in this test. Gupta *et al.* investigated the effect of PVP-K30 on solubility of celecoxib. They showed that by increasing the polymer ratio in solid dispersion of celecoxib, saturation solubility of the drug increased (23). 

*Morphological analysis*

**Figure 1:**
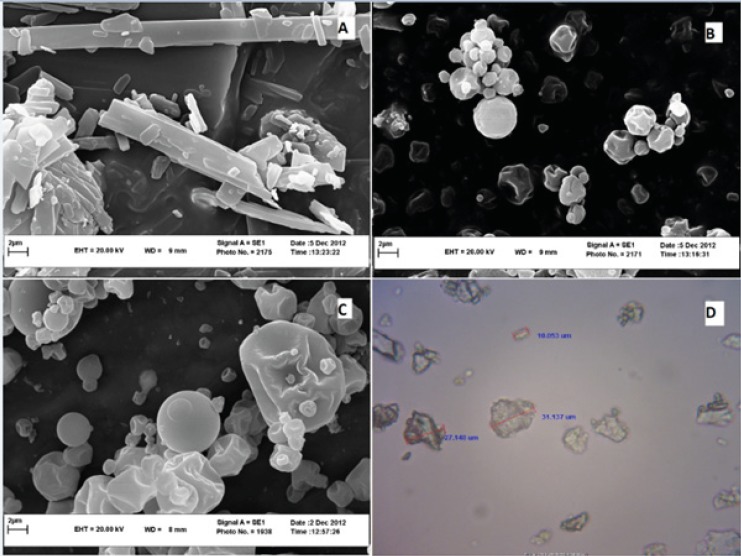
SEM image of pure CLX (A), CLX:SOL 1:1 SD sample (B), CLX:SOL 1:4 SD sample (C) at 10000 magnification, and optical microscope image of sieved fraction of solid dispersion with 1:4 ratio prepared by solvent evaporation method (D) at 1000 magnification

Scanning electron microscopy (SEM) and optical microscopy were used to investigate the morphology of CLX particles. As it was shown in Figure 1A, original CLX showed rod-shaped crystals whilst spray dried samples exhibited spherical particles. Dispersed samples of CLX: SOL with 1:1 ratio were spherical particles with wrinkled surfaces (Figure 1B) whereas by increasing the SOL ratio (CLX: SOL 1:4 SD), spherical particles became larger (Figure 1C). These results confirmed the data obtained in particle size measurements. However, as it was shown in Figure 1D, the 1:4 pulverized samples prepared by solvent evaporation, were irregular in shape. All pulverized samples prepared by solvent evaporation or melting method were similar in shape.

*Differential scanning calorimetry (DSC)*

**Figure 2:**
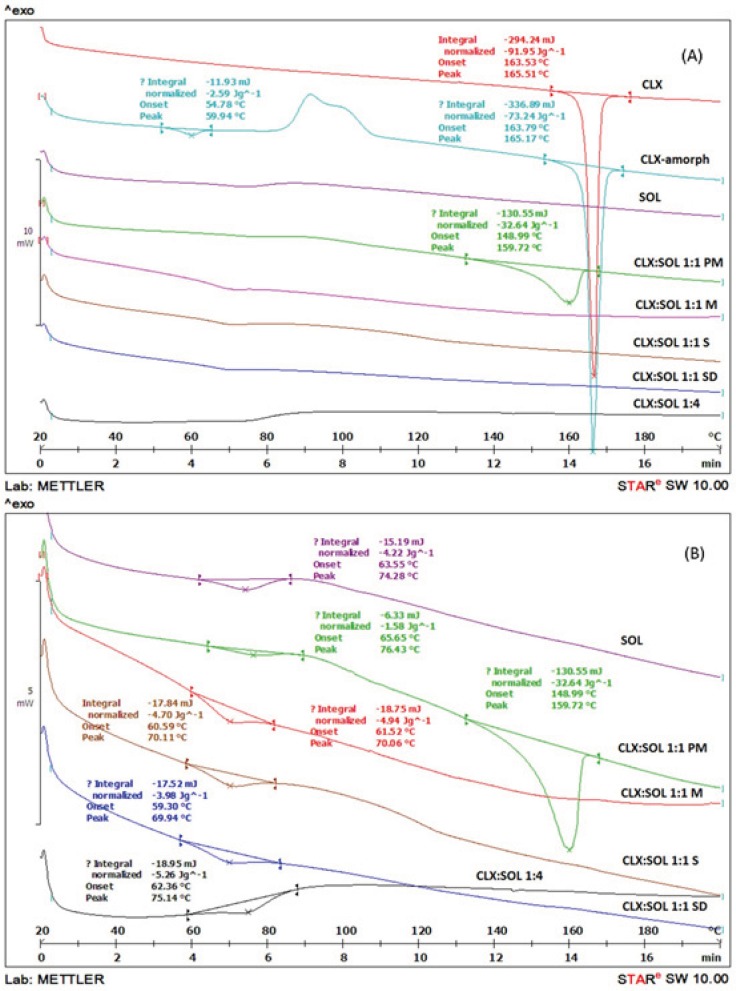
DSC thermograms of pure CLX, SOL, solid dispersion with 1:1 and 1:4 drug:carrier ratio and its physical mixtures in small scale (A) and large scale (B) (PM: Physical mixture, M: Melting method, S: Solvent evaporation method, SD: Spray drying method).

DSC analyses were performed in order to investigate the thermal behavior of the components in the samples. Figure 2 shows DSC thermograms of untreated crystalline CLX, amorphous CLX, SOL and physical mixture and dispersed samples at 1:1 drug: carrier ratio. The DSC scans for all samples with higher amounts of carrier were similar to each other. As an example the DSC scan for dispersed sample at 1:4 drug: carrier ratio prepared by solvent method is shown in Figure 2.

The broad peak of glass transition (T_g_) of pure SOL was detected at 74.28 °C. The onset temperature and sharp endothermic peak for melting point of pure CLX was determined at 163.53 °C and 165.51 °C respectively, with a fusion enthalpy of 91.95 J/g. These results are in agreement with those reported for CLX previously (32). The amorphous CLX exhibited T_g_ at 59.94 °C, a recrystallization exotherm at 72.35 °C and a sharp melting endotherm at 165.17 °C with enthalpy of fusion 73.24 J/g. Physical mixture of CLX: SOL (1:1) showed a broad melting peak from 148.99 °C to 159.72 °C with a fusion enthalpy of 32.64 J/g. This reduction in enthalpy and melting point may be attributed to partial dissolution of CLX in SOL when it was heated above the T_g_ of polymer. Similar findings have been reported previously by other researchers for different polymer-API systems (12, 21, 33). In solid dispersion samples prepared by different methods, all of the DSC thermograms showed a single T_g_ with no noticeable peak for CLX indicating strong interaction between CLX and SOL and changes of CLX to completely amorphous state. In addition there was no difference among the T_g_ of dispersed samples prepared by different methods. As it was shown in Figure 2(B) by increasing the polymer ratio in solid dispersion samples, the T_g_ of binary system has shifted to the higher temperatures *e.g*. the T_g_ of pure amorphous CLX has shifted from 59.94 to 75.14 °C for 1:4 drug to polymer ratio. The increase in T_g_ which limits the molecular mobility of CLX molecules in dispersed samples could account for stabilization of dispersed systems.

 In addition the absences of recrystallization exothermic peak of amorphous CLX in DSC thermograms of dispersed samples indicate that SOL could prevent the recrystallization of CLX when it was heated above the T_g_.

According to Gordon-Taylor equation the theoretical T_gmix_ of dispersed samples were estimated to be 67 and 71 °C for the 1:1 and 1:4 drug to polymer ratio, respectively. However the experimentally determined values were 70 and 75 °C for 1:1 and 1:4 ratios. These positive deviations from ideal behavior could be sign of the intermolecular interaction between CLX and SOL. This interaction is probably due to formation of hydrogen bonding between CLX and SOL as it will be discussed later in IR studies. Such these results have been reported for solid dispersion systems containing PVP and CLX (22) and also other API and carriers (4).

*X-ray powder diffraction studies (XRPD)*

**Figure 3:**
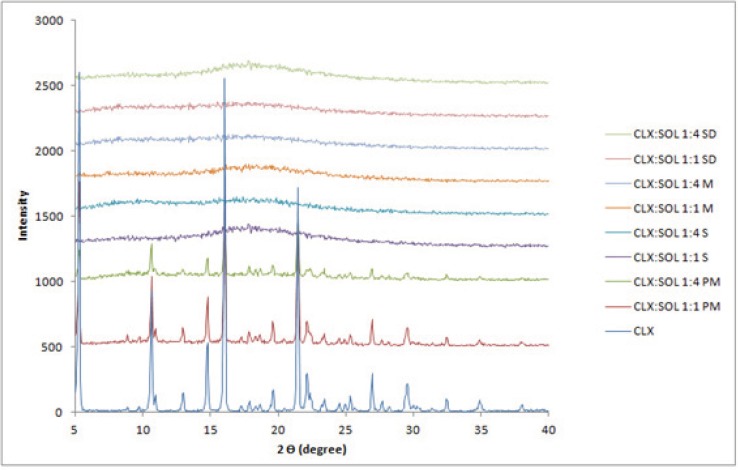
XRPD pattern of pure CLX, SOL, physical mixture and solid dispersion samples with the different ratio. (PM: Physical mixture, M: Melting method, S: Solvent evaporation method, SD: Spray drying).

Further characterization of solid states of samples was carried out by XRPD studies. The patterns obtained for pure CLX, SOL, CLX: SOL physical mixtures and dispersed samples were displayed in Figure 3. The untreated CLX exhibited sharp diffraction peaks at 2θ values of 5.11, 10.94, 16.12 and 21.53 indicating crystalline nature of CLX corresponding to the reported crystal lattice parameters for CLX (32) whilst SOL did not show any peak and exhibited amorphous state (data not shown). The physical mixtures of CLX and SOL showed peak positions similar to CLX, but at lower intensities, indicating that CLX has retained its crystalinity. With increase in the ratio of polymer in physical mixtures (1:4 CLX: SOL), the intensity of peaks decreased. In contrast, no distinct peaks were observed in the diffraction patterns of the dispersed samples even at higher drug: carrier ratio (1:1). The absence of CLX intensive peaks indicates the formation of an amorphous state. These results were in good agreement with DSC studies.

*Fourier transform infrared spectroscopy (FT-IR)*

**Figure 4:**
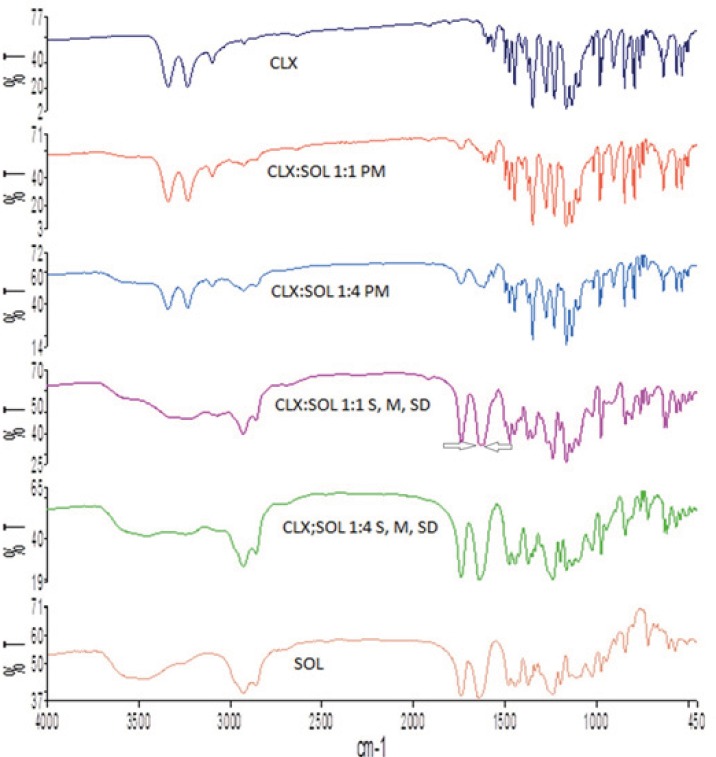
FTIR of pure CLX and SOL, and dispersed samples. (PM: Physical mixture, M: Melting method, S: Solvent evaporation method, SD: Spray drying).

The interaction between API and carrier in different systems usually results in noticeable changes in the FT-IR spectra, so this study was performed to investigate if any interaction has been occurred in dispersed samples at the molecular level. The FT-IR spectra of pure CLX, SOL, their physical mixtures and dispersed samples are shown in Figure 4. The FT-IR spectra of CLX showed characteristic peaks at 3338 and 3234 cm^-1^ that attributed to N-H stretching vibration of SO_2_NH_2_ group, 1348 and 1165 cm^-1^ for the S=O asymmetric and symmetric stretching and 1230 for C-F stretching (32). Spectra of SOL, showed inter-molecularly hydrogen bonded–OH stretching in the 3350–3600 cm^-1^ range, ester carbonyl stretching at 1739 cm^-1^ and C=O stretching for tertiary amide at 1643 cm^-1^. In the FT-IR spectra of physical mixture the double peaks of N-H stretching were slightly weaker whilst in dispersed samples with 1:1 drug:carrier these doublet peaks are weaker and broader. With increase in the amount of SOL (1:4 CLX: SOL) the NH_2_ stretching bands shifted to higher wavenumber and almost diminished. In addition the C=O stretching vibration bond of SOL shifted to lower wavenumber for 1:1 and 1:4 drug: carrier ratios. As it was shown in Figure 3 in CLX:SOL dispersed samples prepared by three different methods, C=O stretching bond appeared in 1637 cm^-1^ and 1641 cm^-1^, for 1:1 and 1:4 drug: carrier ratios respectively. These changes indicate the possibility of hydrogen bonding between the acidic hydrogen of the N-H group of CLX as the hydrogen donor and the C=O group of SOL as the hydrogen acceptor in dispersed samples. Similar results have been reported previously for CLX and other polymers (21, 22). As it was shown in Figure 4 the wavenumber of C=O group of SOL in CLX:SOL 1:1 dispersed samples, appeared as a shoulder at 1637 cm^-1^, Whilst the peak related to C=O of SOL was observed as a sharp peak at 1643 cm^-1^. By increasing the SOL ratio this shift change to 1641 cm^-1^ that is closer to original C=O stretching vibration bond of SOL (1643 cm^-1^). This phenomena could be attributed to the greater number of unbounded C=O groups of SOL molecules by increasing the ratio of SOL in dispersed samples as reported previously for CLX and PVP-K30 (34). 

*Dissolution studies*

**Figure 5:**
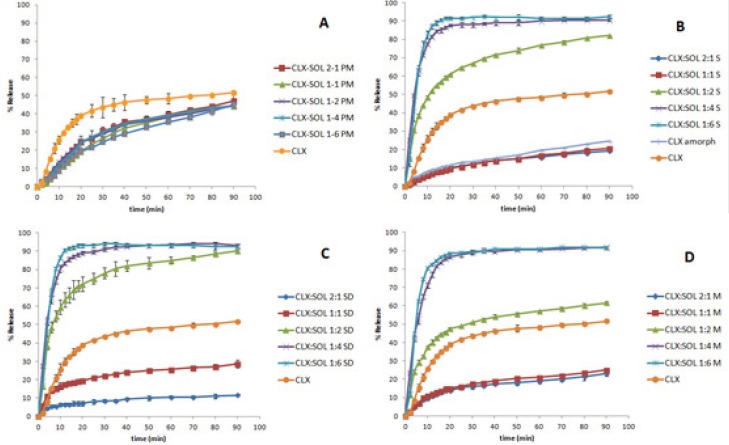
The dissolution profile of pure CLX and its physical mixtures with SOL (A). Its solid dispersions with SOL prepared by solvent evaporation method (B), Spray drying method (C) and by melting method (D) (n=3).

The dissolution behavior of untreated CLX, its physical mixtures, amorphous CLX and dispersed samples prepared by different methods are shown in Figure 5. It is clear that the presence of SOL have a significant effect on dissolution profile of dispersed samples. The dissolution profiles of physical mixtures are presented in Figure 5A. As it was shown the dissolution rate of all physical mixtures were lower than CLX. This could be due to gel formation and the sticky properties of SOL when in contact with water. The results exhibited significant differences among DE and MDT of pure CLX and all PM samples (P < 0.05) whilst there was no significant difference among PM samples. Although the dissolution rate of physical mixtures at the initial times is much lower than pure CLX (*e.g*. 39% of pure CLX dissolved after 20 min whilst this value was between 20-25% for physical mixtures) but at higher dissolution times the dissolution rate of physical mixtures is more facilitated so that at the end of the test, the percent of drug dissolved (50%) was comparable to CLX (52%). 

Interestingly amorphous CLX showed slower dissolution rate than untreated CLX. Slower dissolution rate of amorphous CLX could be attributed to the devitrification of amorphous CLX when in contact to aqueous environment and hence recrystallization to more hydrophobic structure that was reported previously by Puri *et al.* (35). Similar results have been reported for capecitabine (36), felodipine (37) and diazepam (38). In all dispersed systems prepared by different methods, the samples with 2:1 and 1:1 drug to carrier ratios exhibited slower dissolution rate than untreated CLX but comparable to amorphous CLX (statistical analysis showed no significant differences among DE and MDT of 2:1, 1:1 and amorphous CLX), however with increasing the concentration of SOL in the samples (from 1:2 to 1:6) a remarkable enhancement in drug dissolution was observed. These results indicate that the ratio of drug to carrier is one of the main factors controlling the dissolution rate of drugs which is in agreement with other studies (5, 6 and 39). For samples obtained by solvent evaporation methods i.e. conventional and spray drying techniques, (Figure 5B and C) at drug: carrier ratios of 1:4 and 1:6 approximately 90% of drug was released within 20 min, whilst in the case of CLX alone only 39% was dissolved within 20 min. Figure 4D illustrates similar results for samples prepared by melting method. DE and MDT of all samples are presented in Table 1. Generally, the highest DE values were observed at higher concentrations of SOL (drug: carrier ratio of 1:4 or 1:6). There was no significant differences between DE and MDT of all 1:4 and 1:6 dispersed samples (P < 0.05) hence the method of preparation had no remarkable effect on the dissolution profiles of 1:4 and 1:6 samples (Figure 5). 

As it was shown in Figure 5 the dissolution rate of dispersed samples with the ratio of 2:1 and 1:1 CLX: SOL was slower compared to CLX and was similar to amorphous CLX. At low amount of carrier in dispersed samples, amorphous CLX are probably present as aggregates or clusters due to their lipophilicity. This corresponds to glass suspension type of solid dispersion systems (40). Therefore the less available carrier to surround drug particles individually, and also formation of the viscous gel around CLX clusters could delay the hydration of drug particles and consequently retard the dissolution rate. With increasing the ratio of carrier (1:4 and 1:6), the excess amount of SOL could results in molecularly dispersion of drug particles within carrier and therefore better wettability of drug when in contact with the dissolution medium. Similar results were reported for solid dispersion of CLX and HPMC at lower amount of HPMC (41). Van Drooge *et al.* also reported a similar result when examining the slow-dissolution behavior of diazepam from a disaccharide glass based solid dispersion (38).

As it was shown in Figure 5B and C, the solvent evaporation methods particularly by spray drying technique could be more effective to enhance the dissolution rate of drug. For example at 1:2 CLX:SOL, 48% of drug released after 20 min from dispersed samples prepared by melting method whilst this value was 61% and 72% for samples prepared by conventional solvent evaporation method and spray drying techniques respectively. This may be attributed to the smaller mean particle size of powder obtained by spray drying techniques (Table 1). On the other hand, the method of preparation of dispersed systems plays a critical role only in the dissolution profile of 1:2 CLX: SOL samples whilst in samples with the higher ratio of carrier, method of preparation did not have any remarkable effect on the dissolution rate. Generally it can be concluded that at high concentration of SOL, carrier mostly controls the dissolution rate of the drug, but at 1:2 ratio of CLX: SOL physicochemical properties of particles *e.g*. particle size is also important along with the effect of hydrophilic carrier (42).

*Long term stability*

**Figure 6:**
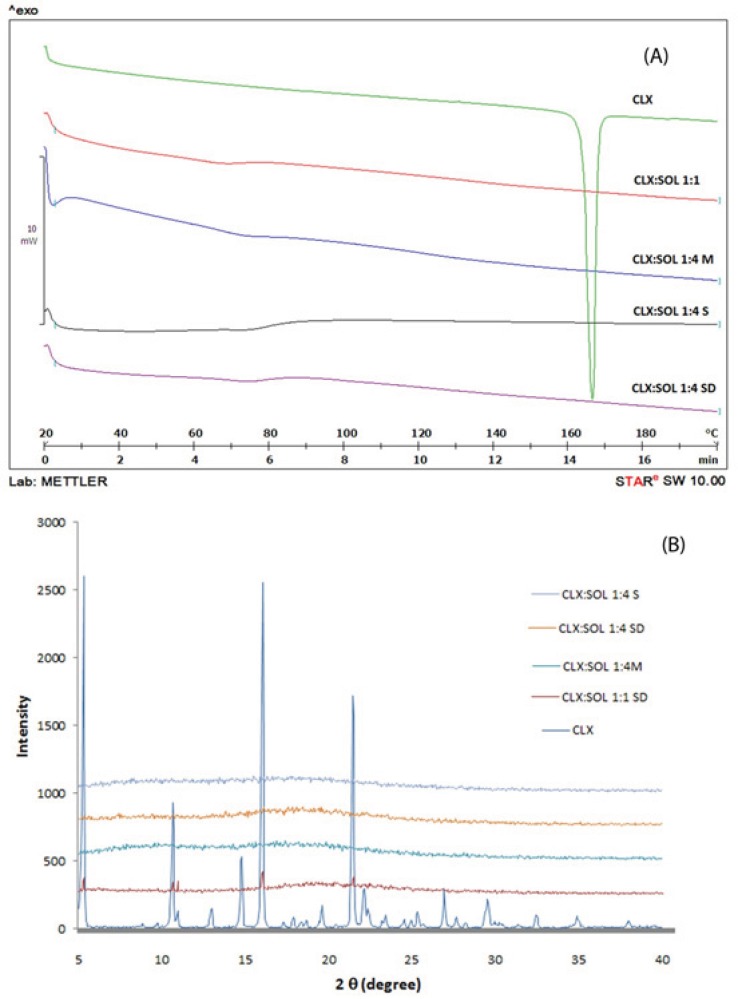
DSC thermograms and XRPD pattern of pure CLX and solid dispersions with 1:1 and 1:4 drug:carrier ratio after 12 months storage at ambient condition. (M: Melting method, S: Solvent evaporation method, SD: Spray drying method).

Amorphous systems are thermodynamically unstable and have a more tendency to convert to stable (crystal) form. Recrystallization of amorphous drug is the main disadvantage of solid dispersion systems. Any change in crystalinity of the drug could be reflected in DSC, XRPD pattern and/or dissolution profiles. The DSC thermograms and XRPD patterns of 1:1 and 1:4 drug: carrier samples stored at ambient condition for 12 months are shown in Figure 6(A) and (B). These figures clearly indicate that there are no remarkable changes in the DSC thermograms and XRPD patterns of aged 1:4 samples compared to newly prepared ones (Figure 2 and 3) indicating that the amorphous nature of the drug has been reserved after storage at ambient conditions. However some small peaks appeared at 5, 16 and 21 2 values for 1:1 drug: polymer ratio (Figure 6(B)). These finding indicate that at high SOL ratios, polymer could stabilize amorphous nature of CLX in dispersed samples and hence SOL could inhibit recrystallization of CLX during storage over one year. 

**Figure 7:**
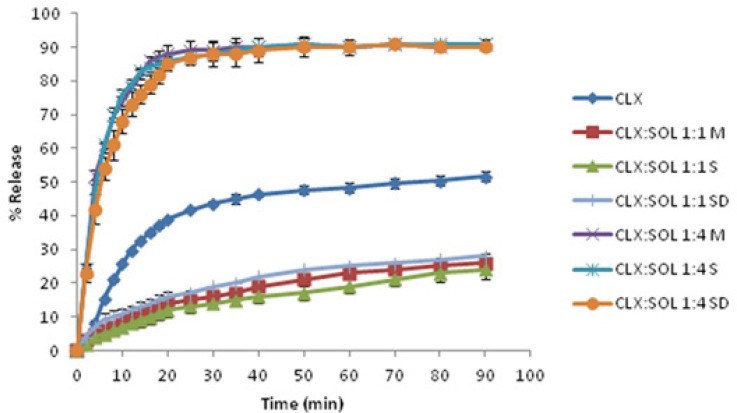
The dissolution profiles of pure CLX and its solid dispersions with SOL after 12 months storage at ambient condition. (M: Melting method, S: Solvent evaporation method, SD: Spray drying method) (n=3).

Figure 7 shows the dissolution profiles of samples stored at ambient conditions. The comparison of dissolution profiles of these samples with newly prepared ones (Figure 5) and also the values for *f*_1 _(<15) and *f*_2_ (>50) indicate that there is no considerable change in drug dissolution after 1 year.

## Conclusions

It could be concluded that the use of SOL in dispersed formulations can enhance the dissolution rate of poorly water-soluble drug, CLX. At high SOL content (1:4 or 1:6) the rate of dissolution enhancement was mostly dependent on the ratio of drug: carrier and not the method of dispersion preparation. However at 1:2 ratio the method of dispersion preparation has also contributed to the dissolution of CLX and the spray dried samples exhibited higher dissolution rate than corresponding samples prepared by other methods. It was shown that with increase in SOL ratio in dispersed samples, the solubility of CLX increased. DSC and XRPD analyses confirmed the presence of amorphous state of CLX in dispersed systems. FTIR studies indicated the formation of hydrogen bonding between CLX and SOL. The long term stability studies showed that SOL could prevent the recrystallization of CLX in dispersed samples. The results of this study showed that SOL is a suitable carrier to enhance dissolution rate of CLX in dispersed formulations.
